# Evaluating antibacterial and antioxidant properties of sericin recovered from cocoons of *Bombyx mori*, *Gonometa postica* and *Samia ricini* in Kenya

**DOI:** 10.1371/journal.pone.0316259

**Published:** 2024-12-31

**Authors:** Mwangi G. Kanyora, Timothy M. Kegode, Justus Kurgat, Harrison Kibogo, George Asudi, Chrysantus M. Tanga, Workneh Ayalew, Subramanian Sevgan, Nelly Ndungu

**Affiliations:** 1 International Centre of Insect Physiology and Ecology (*icipe*), Nairobi, Kenya; 2 Department of Biochemistry, Microbiology and Biotechnology Kenyatta University, Nairobi, Kenya; 3 Department of Biochemistry, Jomo Kenyatta University of Agriculture and Technology, Nairobi, Kenya; University of Waterloo, CANADA

## Abstract

Microbial infections and excessive reactive oxygen species are the primary contributors to delays in wound healing with *Pseudomonas aeruginosa*, *Escherichia coli* and *Staphylococcus aureus* as the common wound infection causing bacteria. In fact, wound management has become more challenging since most of these microbes have developed resistance against commonly used conventional antibiotics thus making it necessary to develop natural products with both antibacterial and antioxidant activities. Increasing attention has been paid to silk sericin in the last decade, with limited research focus in Africa. Therefore, this work focus on evaluating antibacterial and antioxidant capacity of sericin recovered from cocoons of domesticated (*Bombyx mori*, *Samia ricini*) and wild (*Gonometa postica*) silkworms in Kenya. Sericin recovery was achieved using high temperature-high pressure method. Results revealed significance interspecies variation in all the parameters. Total flavonoid content ranged between 270±60.1 and 603.3±44.1 mg GAE/100g with *S*. *ricini* demonstrating the highest whereas *G*. *postica* exhibited the least content. Moreover, *S*. *ricini* showed the highest total phenolic content at 780.0±67.6 mg QE/100g while *G*. *postica* had the least phenolic content at 330.6±14.6 mg QE/100g. *Samia ricini* revealed the highest radical scavenging capacity at 40.47 ± 3.76% whereas *B*. *mori* sericin extract showed the least radical scavenging ability at 24.6± 2.96%. Furthermore, *S*. *ricini* silk sericin extract demonstrated the highest inhibitory activity against *Staphylococcus aureus*, *Pseudomonas aeruginosa* and *Klebsiella pneumonia* which translated to 70.79 ± 11.28%, 93.86 ± 1.92%, 94.77 ± 7.07% when compared to streptomycin, chloramphenicol and oxytetracycline respectively. *Bombyx mori* and *Gonometa postica* showed the highest inhibitory activity against *S*. *pyogene* and *E*. *coli* respectively. These findings uncovered sufficient antibacterial efficacy of all three silk sericin extracts against both Gram-positive and negative bacteria, however, in depth research is still required to guarantee the aforementioned bioactivities to boost the therapeutic potential of silk sericin-based biomaterials.

## Introduction

Sericin is a natural glycoprotein synthesized in silkworm glands and accounts for 20–30% of the entire cocoon weight [1–3]. It has a molecular weight in the range of 10–400 kDA depending on the temperature, pH, processing time and the recovery method applied [4]. For example, the molecular weights of sericin recovered by urea, acid, alkali and heat degradation methods are 10–225 kDa, 50–150 kDa, 15–75 kDa and 25–150 kDa respectively [2, 5, 6]. Sericin is composed of several amino acids with serine and threonine forming the major percentages [7]. These amino acids possess strong polar groups such as carboxyl, amino and hydroxyl group that allow sericin to form crosslinks, copolymerize and interact with other polymers [2, 8, 9]. Over many decades, the high organic wastewater obtained during the degumming processes has always been discarded [10, 11]. Global statistics indicate that out of the 400,000 tons of cocoon generated worldwide, 50,000 tons of sericin is never utilized [12–14]. However, recent findings indicate that sericin possess desirable biological activities [7, 15–17] which has increased interest for its recovery from degumming wastewaters, cocoons and even from silk glands for its application in food [3] cosmetic [18] and pharmaceutical industries [2, 6, 19]. However, on this study the focus was on evaluating the antibacterial activity of sericin against the most common bacteria that impedes wound healing [20]. Further, given that persistence wound infections is associated with prolonged inflammation phase characterized by oxidative stress [21], this study also evaluated the antioxidant capacity of silk sericin extracts from the three species.

One key clinical problem that substantially impedes wound healing is wound infection [22]. Anti-infection is crucial especially while treating certain chronic wounds such as pressure ulcers, diabetes and vascular injuries [23]. According to earlier research, most wounds are infected with polymicrobial infections, which are mostly caused by aerobic and anaerobic bacteria with trace amounts of fungi and viruses [24, 25]. Bacterial infections of wounds cause increased exudation and hinder the growth of granulation tissue at the wound site [20]. In addition to reducing collagen deposition, bacterial endotoxins can cause a sustained elevation of pro-inflammatory cytokines including TNF-α and IL-1β, which in turn decreases the production of growth factors [26]. During the initial phase of wound infections, *P*. *aeruginosa*, *S*. *aureus*, *K*. *pneumoniae*, *Enterococcus faecalis* and *Acinetobacter baumannii* are the most frequent bacterial species that invade and cause infections to the wound with *S*. *aureus* being the most frequent invaders. Later, *Pseudomonas aeruginosa* starts to colonize wounds and causes sepsis upon getting inside the lymphatic and blood arteries [27, 28].

In a recent study on wound microbial isolation and accessing the antimicrobial resistance, 29.2% of the isolated strains showed resistance against 6 drugs. Out of these strains, S. *aureus* and *P*. *aeruginosa* formed the majority while *E*. *coli*, *K*. *pneumoniae*, *P*. *mirabilis* and *A*. *baumannii closely* followed [29] Therefore, for wounds to heal effectively and efficiently, pathogenic bacterial growth at wound sites should be inhibited. Reactive oxygen species (ROS) are important modulators of several phases of wound healing with their low levels being necessary to prevent external injury [30]. Wound deterioration is primarily caused by redox imbalances, resulting from increased oxidative stress on tissues and a decline in antioxidant capacity [21, 31, 32]. Wound dressings must include both antibacterial and antioxidant properties in order to prevent bacterial infections and control the excessive formation of ROS at the wound site [33].

The skin acts as a primary barrier against pathogen colonization [34] and when the normal anatomical structure of the skin is disrupted by surgery, physical, chemical or mechanical trauma it results into a wound [35]. Based on pathogenesis and consequences, wounds are classified as either acute or chronic. Acute wounds heal faster and the structural integrity being gradually restored [36], while chronic wounds fail to follow the normal wound healing processes. Chronic wounds are brought about by internal variables that may be linked to lifestyle illnesses such as diabetes and hypertension [37]. Wound healing is a sequential process which includes; hemostasis, inflammation, proliferation, and remodeling [38, 39]. The initial phase of wound healing entails the application of strategies to reduce blood loss through vasoconstriction and the formation of blood clots [38, 40]. Immune system cells, including neutrophils, lymphocytes, macrophages and signaling molecules do intricately coordinate to cause inflammation throughout the healing process, this phase usually begins after hemostasis. As the predominant leukocytes in wounds, neutrophils are essential in the fight against infection using multiple defense mechanisms, including proteases, antimicrobial peptides and ROS [41]. These cells have finite life span and undergo programmed cell death via mechanisms involving caspases and cathepsin D [42]. Proliferative stage entails a complex process which includes neovascularization, re-epithelialization, granulation tissue formation and immune system modulation while remodeling phase entails neo-vasculature regression and transformation of granulation tissue into scar tissue [43, 44].

Numerous bioactivities of silk sericin have been shown in earlier investigations suggesting that it could act as a potential source of antimicrobial agents for application in wound management [7, 45–48]. However, there is little or no information regarding the antibacterial and antioxidant activity of sericin recovered from Africa and specifically Kenya. Therefore, the objective of this study was to determine the antioxidant and antibacterial activity of sericin recovered from cocoons of *B*. *mori*, *S*. *ricini* and *G*. *postica* in Kenya. We performed antibacterial assays against *E*. *coli*, *K*. *pneumonia*, *P*. *aeruginosa*, *S*. *aureus* and *S*. *pyogene*.

## Materials and methods

### Sample preparation and sericin recovery

*Bombyx mori* and *S*. *ricini* were collected from the International Centre of Insect Physiology and Ecology (*icipe*) sericulture rearing unit in Nairobi-Kenya (1° 13’ S, 36° 53 E). *Gonometa postica* cocoons on the other hand were collected from Mumoni forests situated in Kitui County, Eastern Kenya (0° 31’ 29" S, 37° 58’ 47" E). This study applied the higher temperature-high pressure (HTHP) method for sericin recovery due to its reduced recovery time, low cost, high efficiency, simplicity, environmental friendliness and lack of a purification step after recovery [49]. Sericin recovery was achieved as previously established by Hossain *et al*.,2023 and Aramwit *et al*.,2019 [47, 50] with minor modifications. In brief, cocoons were cut into smaller pieces (approximately 5 mm^2^), washed with distilled water and dried overnight at 60°C. Approximately 5g of cocoons were added to 250 ml deionized H_2_O and autoclaved for 60 min at 120°C and 15 lbf/in^2^. The solution was filtered using Whatman No 1 filter paper to remove fibroin and any other undissolved materials followed by cold ethanol precipitation in the ratio of 1:1 v/v. Following a 20-minute centrifugation at 4,200 rpm, the filtrate was frozen at -80°C overnight. Subsequently, the filtrate was freeze-dried at -80°C for 24 hours to obtain sericin powder.

The percentage recovery yield of sericin was calculated with the following weight method formulae:

YExtraction(%)=(WSP/WC)˟100,

Y _Extraction_ refers to sericin extraction yield,

W_SP_ refers to sericin powder dry weight and W_C_ is the initial cocoon dry weight.

### Total Phenolic Content (TPC)

Total phenolic content in sericin extracts was evaluated using the Folin-Ciocalteu colorimetric assay as described by Butkhup *et al*,.[51] with minor modifications. Gallic acid (GA) was chosen as a standard based on its purity and stability. Briefly, 5 mL of 0.2 N Folin–Ciocalteu reagent was mixed with 1 mL of sample extract (10 mg/ml) and followed by an additional 4 mL of 75 g/L sodium carbonate, after 5 min. The mixture was placed in the dark at room temperature for 2 h after which the absorbance readings were read at 760 nm against a blank solution using a spectrophotometer. About 1 ml of gallic acid prepared in different concentrations (0–250 μg/mL) was added in 5 mL of 0.2 N Folin–Chicalote instead of the sample, after 5 minutes, 4 mL of 75 g/L sodium carbonate were added to the mixture. The mixture was subjected to a dark place for 2 hours after which the absorbance was read at 760 nm. Gallic acid (standard) was used to generate a standard calibration curve (y = 0.0073x + 0.0233, *R*^2^ = 0.999). The samples’ absorbance readings were compared to the gallic acid standard to determine the sample’s phenolic concentrations. The total phenolic content results were expressed as mg of gallic acid equivalent present in 100 g of silk sericin extracts (mg GAE/100 g sericin), where GAE represents Gallic acid equivalent.

### Total Flavonoids Content (TFC)

The flavonoid content was determined in 10 mg/ml of silk sericin extract using Aluminum chloride (AlCl_3_) in a calorimetric assay as earlier described by Kegode *et al*,.[52] with minor modifications. Briefly, 4 ml distilled water was added to 1 ml of silk sericin extract (10 mg/ml) before mixing with 0.3 ml of 5% (w/v) NaNO_2_. The mixture was left to stand for 5 minutes after which 0.3 mL of 10% AlCl_3_ was added. After 1 minute, 2 mL of 1 M NaOH was gradually added to the mixture followed by an additional 2.4 ml of distilled H_2_O to mark the final step. Absorbance measurements were obtained at 510 nm using a spectrophotometer against a blank. The flavonoid concentration in the sericin samples was determined by comparing the absorbance measurements to the standard calibration curve (y = 0.0006x + 0.0028, *R*^2^ = 0.9981) generated using 20–200 μg/mL quercetin. The total flavonoid content results were expressed as mg of quercetin equivalent present in 100 g of silk sericin extracts (mg QE/100 g sericin).

### 2,2-diphenyl-1-picrylhydrazyl (DPPH) free radical scavenging ability

The antioxidant activity of the sericin extract was assessed using DPPH, a relatively stable free radical that could be reduced especially by more powerful reducing agents such as phenolic compounds [53] following a protocol established previously by Kegode *et al* [54] with minor modifications. DPPH assay is the most widely applied method for determining the scavenging ability of samples [53, 55]. The method is based on the ability of DPPH radical to react with all compounds capable of donating an electron [56]. DPPH molecules give maximum absorbance readings at 517nm and are violet in color under stable conditions but upon reacting with any reducing agent such as sericin its color changes to pale yellow alongside a reduction in absorbance readings [56]. In brief, 0.5 ml of sericin (40mg/ml) solution was mixed with 3 ml of DPPH-methanolic solution (2mg/100 ml methanol), kept in darkness for 60 min. The antioxidant activity of sericin was determined by measuring the absorbance of the mixture at 517 nm using a spectrophotometer. In place of sericin, methanol was used as a control. Each sample was assayed in triplicates and the percentage of DPPH radical scavenging activity was calculated using the following formulae;

AntioxidantActivity(%)=[(controlabsorbance–sampleabsorbance/Controlabsorbance]×100

### Antibacterial activity of sericin

#### Bacteria inoculum preparation

Gram-negative (*E*. *coli* ATCC 25922, *K*. *pneumonia* ATCC 13883, and *P*. *aeruginosa* ATCC 27853) and Gram- positive *S*. *aureus* ATCC 25923 were cultured overnight on Muller Hinton Agar (MHA) while *S*. *pyogene* ATCC 19615 (Gram-positive) was cultured on Blood Agar (BA). Single colonies of each bacteria colony were inoculated in sterile H_2_O to achieve a turbidity of 0.5 Mc Farland equivalent to 1×10^8^ CFU ml−1 as recommended by clinical and laboratory standards institute. This was achieved by measuring the optical density (OD) of 0.132 at a wavelength of 600 nm.

#### Disc diffusion assay

Disc diffusion protocol was applied for this study as published in [57]. Briefly, sterile Petri dishes with a diameter of 90 mm containing 25 ml of sterile MHA and BA were inoculated with 100 μL of bacteria sourced from the overnight cultures. For even distribution of bacteria, 10 zirconia beads were used. Sericin stock solution (20mg/ml) was prepared using sterile H_2_O. For every Petri dish, four circular filter discs measuring 6 mm in diameter were placed on the agar-containing bacteria under test and impregnated with 50 μl of silk sericin extract solution. The Petri dishes were then incubated at 37°C overnight after which the diameter of inhibition zones was measured using Image J software. Streptomycin (10 μg), oxy-tetracycline (30 μg) and chloramphenicol (50 μg) discs were used as positive controls while sterile water was used to act as a negative control. Triplicate analyses were performed on each sample and the average determined.

### Statistical analysis

Kruskal–Walli’s test was used to analyze the variations in total phenolic and flavonoid contents of the SS extracts at *P*< 0.05 after confirming that the data was not normally distributed. Dunn’s test was then carried out for pairwise comparisons between individual species. The correlation between total phenolic content (TPC), total flavonoid content (TFC) and antioxidant was investigated using spearman’s rank correlation coefficient. All analyses were done using R studio (v3.5.0) for R (R Core Team 2019).

## Results

### Silk sericin yield

Sericin yields from the entire cocoon weight varied significantly across the three species (*P* = 0.0001). *Bombyx mori* cocoon had the highest yield at 29.87 ± 11.29% of the entire cocoon weight followed by *G*. *postica* at 11.02 ± 1.03% while *S*. *ricini* cocoon had the least percentage recovery yield at 5.50 ± 2.75%.

### Total phenol and total flavonoid content of sericin

The total phenolic and flavonoid contents of sericin varied significantly across the species at (*P* = 0.0391) and (*P* = 0.0258) respectively. *Samia ricini* had the highest phenolic contents at 780.0±67.6 mg GAE/100 g as well as the highest flavonoid content at 603.3 ±44.1 mg QE/100 g. Conversely, *G*. *postica* exhibited the least phenolic content at 287.7±47.5 mg GAE/100 g and the least flavonoid content at 231.1±19.2 mg QE/100 g (Table 1). Interestingly, regarding these two phytochemicals, it’s important to note that all the three species had higher phenolic contents than flavonoid contents.

**Table 1:** Total phenolic and flavonoid content (mean ± SD) of sericin.

Silkworm species (cocoon)	Total phenolic content	Total flavonoid content
	(mg GAE/100 g)	(mg QE/100 g)
***Bombyx mori***	330.6±14.6^a^	270±60.1^a^
***Gonometa postica***	287.7±47.5^a^	231.1±19.2^a^
***Samia ricini***	780.0±67.6^b^	603.3±44.1^b^
***P*-value**	*P* ≤ 0.0391	*P* ≤ 0.0258

Different superscript letters indicate individual variations among the species.

**Fig 1:**
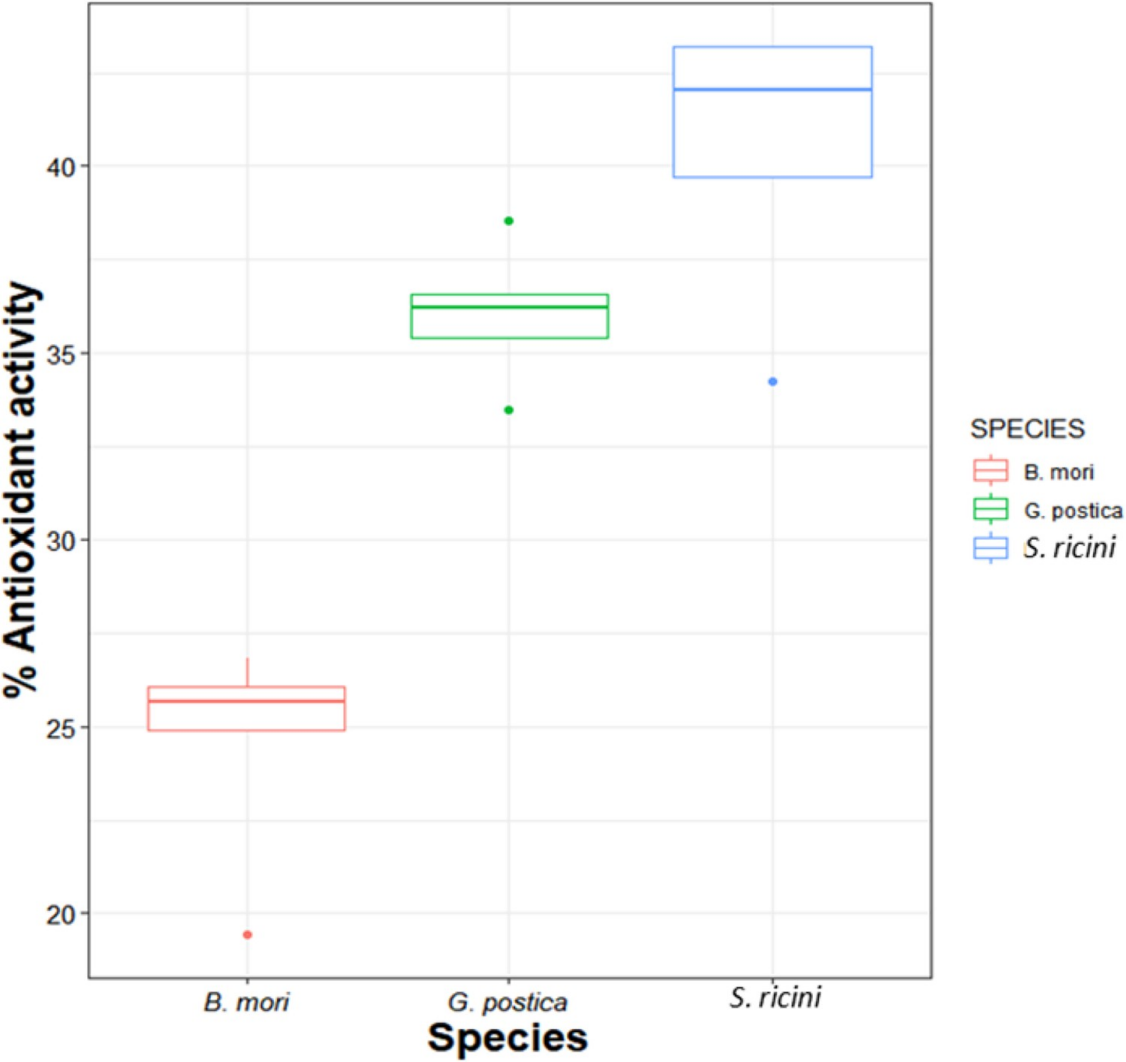
Antioxidant percentages mean of the three SS extracts recovered from cocoons of three distance silkworm species in Kenya.

We deduced a significant positive correlation between phytochemicals and the antioxidant activity of sericin. The correlation between TPC and Antioxidant activity was higher (r_s_ = 0.71) while the correlation between TFC and Antioxidant activity was lower (r_s_ = 0.69) as indicated in Fig 1 below.

### Antioxidant activity assay

#### Radical scavenging activity (DPPH)

**Fig 2:**
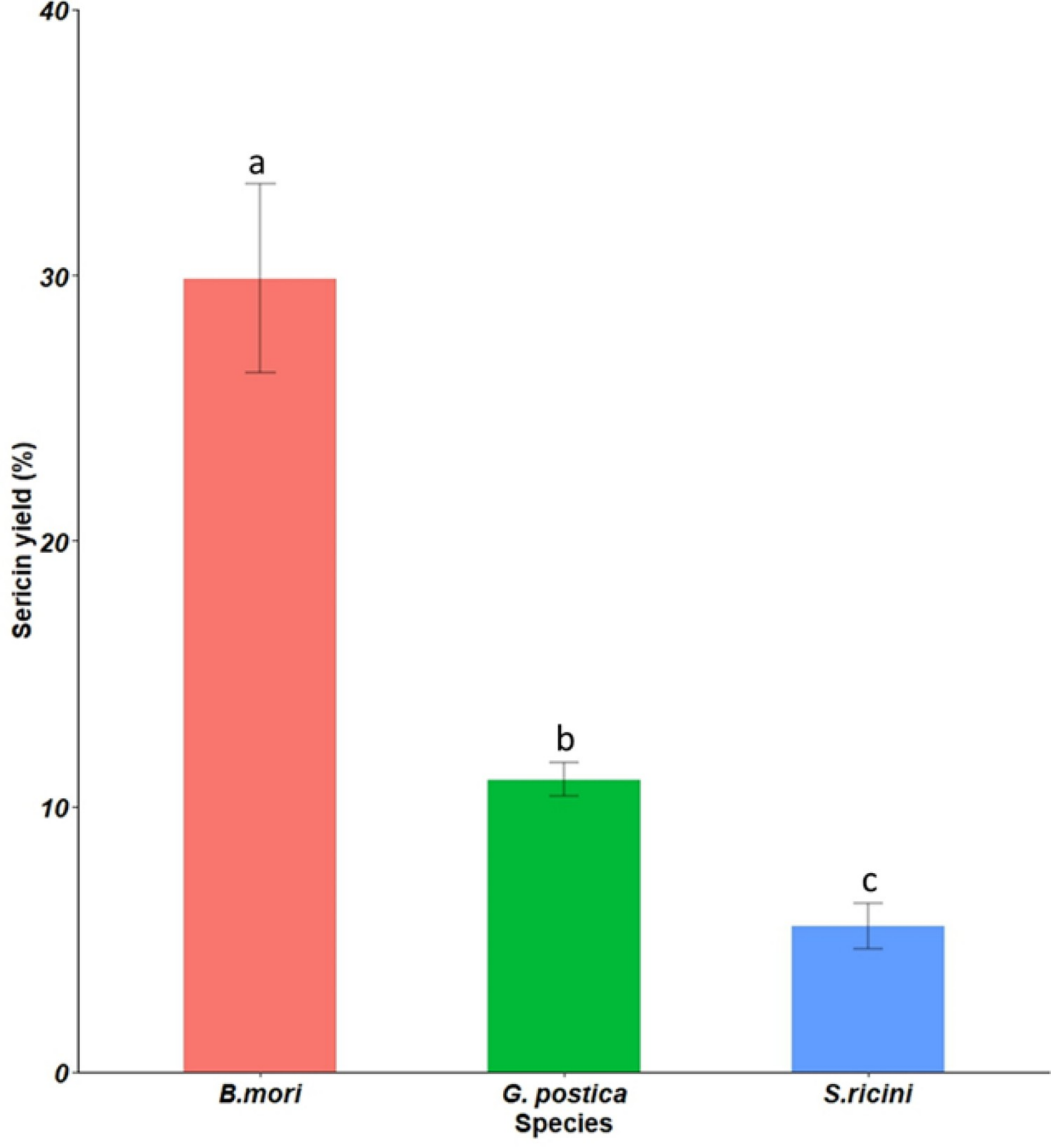
Mean percentages of sericin yields recovered from cocoons of the three distinct silkworms.

**Fig 3:**
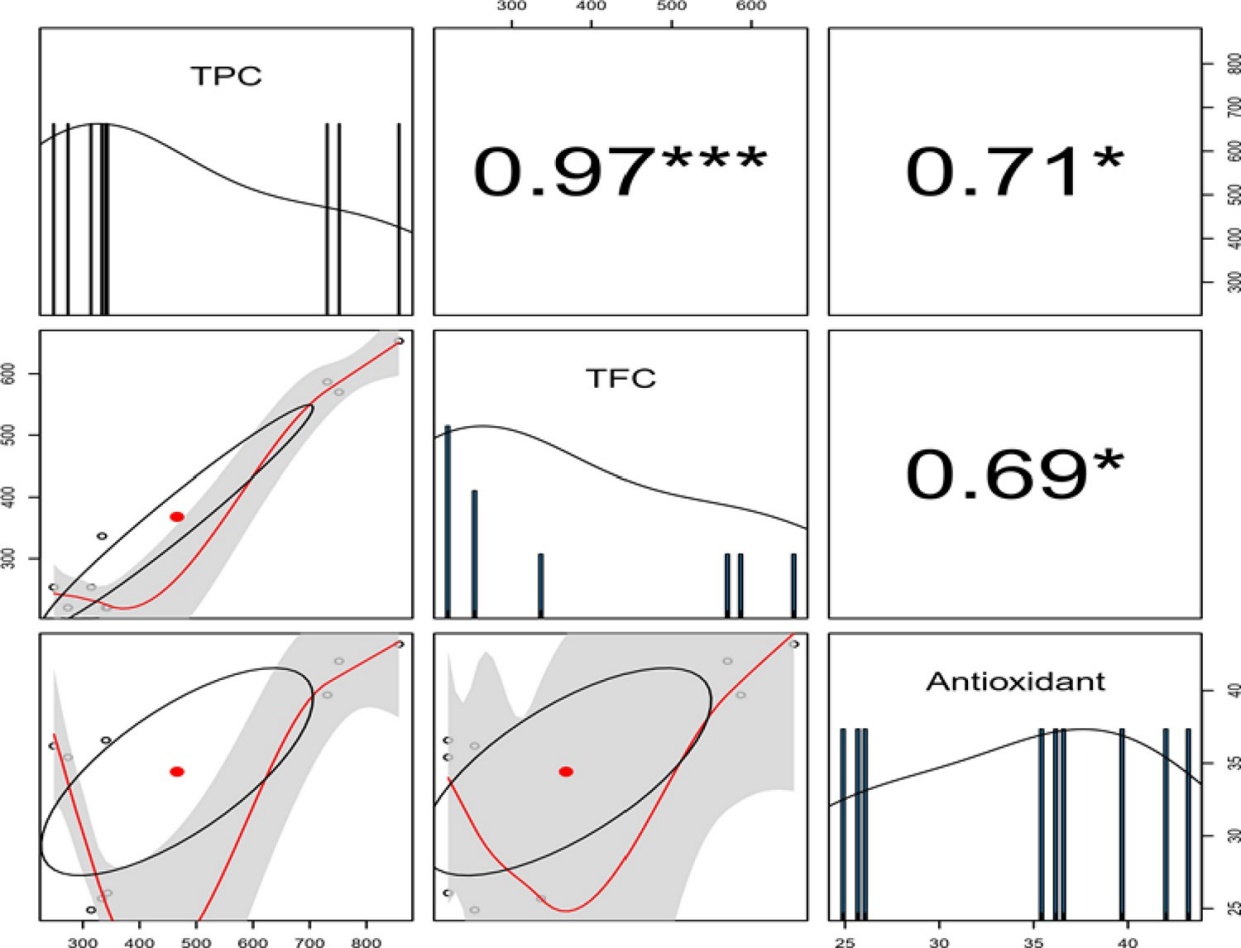
Relationship between total flavonoid content (TFC), total phenolic content (TPC) and antioxidant activity of sericin ***, * indicates significance level at p = 0.001 and p = 0.05 respectively.

Sericin from all the silkworm species exhibited considerable antioxidant activity which varied across the three species (*P* = 0.0001). *Samia ricini* SS extract exhibited the highest radical scavenging activity at 40.5 ± 3.76% followed *G*. *postica* at 36.03 ± 1.84% while *B*. *mori* had the least radical scavenging activity at 24.6± 2.96% (Figs 2 and 3).

#### Antibacterial activity of sericin

Sericin from all the species demonstrated broad-spectrum antibacterial activity. We measured the activity in relation to the conventional antibiotics against the bacteria and presented the results as percentage of the activity exhibited. This was arrived at using the formulae:

Inhibition(%)=sericinzoneofinhibition/Antibioticzoneofinhibtion*100

**Fig 4:**
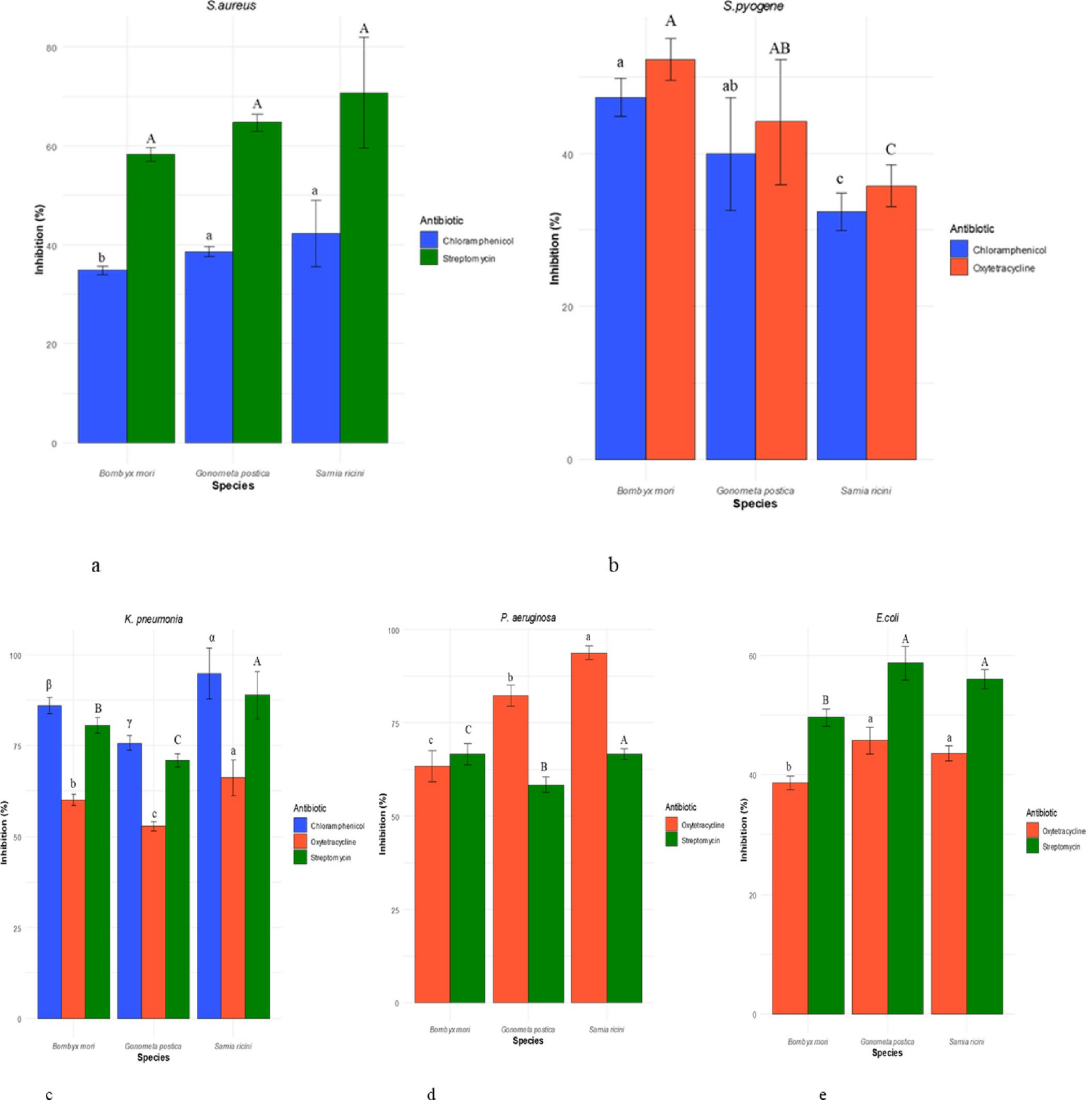
Percentage inhibition zones of the three silk sericin extracts in relation to conventional antibiotics against two Gram-positive (Fig 4A & 4B) and three Gram-negative (Fig 4c, 4d & 4e) bacteria.

S. *ricini* demonstrated the best activity against *S*. *aureus* at 70.79 ± 11.28% that of streptomycin (10 μg). However, there was no significant difference (*P* = 0.152) between the species (Fig 4A). Sericin antibacterial activity against *S*. *pyogene* varied significantly across the three species (*P* = 0.0229) with *B*. *mori* showing the highest activity at 47.39 ± 2.49% and 52.33 ± 2.75% that of chloramphenicol (50 μg) and oxytetracycline (30 μg) respectively (Fig 4B). Additionally, the sericin activity on *K*. *pneumoniae* differed across the three species for chloramphenicol (*P =* 0.0012), oxytetracycline (*P =* 0.0055) and streptomycin (*P =* 0.0055). *Samia ricini* had the highest effectiveness against *K*. *pneumoniae* which was at 93.20 ± 4.34%, 66.20 ± 4.94% and 88.83 ± 6.62% that of chloramphenicol, oxytetracycline and streptomycin respectively (Fig 4C). Further, the effectiveness of silk sericin against *P*. *aeruginosa* varied (*P* = 0.0001) across the three species. S. *ricini* exhibited the best activity which was at 93.86 ± 1.92% and 66.63 ± 1.36% that of oxytetracycline and streptomycin respectively (Fig 4D). Finally, the antibacterial activity of sericin against *E*. *coli* varied significantly at *P* = 0.0044 with *G*. *postica* being the most effective at 45.70 ± 2.19% and 58.65 ± 2.81% that of oxytetracycline and streptomycin respectively. Even so, there was no significant difference between *G*. *postica* and S. *ricini* (Fig 4E).

## Discussion

### Sericin yields

All the species had considerable amount of sericin recovered from their cocoons. This natural protein has the role of gluing the fibroin fibers together in the cocoons [2, 7]. We applied the HTHP degumming method which is greatly influenced by sericin ability to dissolve in hot water [5, 50]. High temperature and pressure permit water to interact with polar amino acids hydroxyl groups thus promoting the separation of silk sericin from fibroin filaments [58]. In our results, *B*. *mori* yielded the highest sericin percentage recovery which was higher than that obtained from four *B*. *mori* silkworm strains using the HTPT method in Thailand ranging from 17–21% [50]. In a different study, Hossain *et al* [47] recorded a higher sericin percentage recovery value at 33% from the cocoons of *B*. *mori* race 4 in Bangladesh using HTHP method (110°C, 30 min). Their work demonstrated that silk sericin yields could be influenced by silkworms strain, recovery duration temperatures [47].

The findings of this study on the percentage recovery of sericin from non-mulberry silkworm cocoons fall between the previously reported ranges of 6–11% [59]. Low levels of sericin in non- mulberry silkworms could be attributed to several issues including environmental variations, differences in their genetic make-up, species and their respective host plants. polyphagous silkworms for instance are linked with to increased degumming losses values [60]. Moreover, sericin yields in non-mulberry plants could be linked to the status of the cocoon, type of crop (multi, tri, and bi-voltine) and the location of harvesting. Furthermore, because the majority of non-mulberry silkworms are wild, their cocoons may have extremely high levels of impurities due to their unfavorable environments [61].

### Total phenolic and flavonoid content

Polyphenols are secondary metabolites normally secreted by plants and are known to have an array of biochemical activities including anti-tyrosinase, anti-carcinogenic and antioxidant [62]. In this study, all silkworm species had considerable amounts of flavonoids and phenolic compounds. As was the case in our investigation, Butkhup and co- authors [51] established that the three *B*. *mori* strains and the Eri silkworm silk sericin extracts in Thailand had higher total phenolic content than total flavonoid content. TPC for *B*. *mori* strains ranged from 393 to 532 mg GAE / 100g, thus our value (330 mg GAE / 100g) was marginally lower, TPC for Eri silkworm was 266 mg GAE / 100g, which is enormously low in comparison to our value of 780 mg GAE/100g [51]. The non-protein components in the cocoons are mostly derived from these secondary metabolites, which are consumed by silkworms. These plant-produced metabolites serve essential defensive and protective functions that improve the host plant’s chances of surviving and reproducing, even though they do not directly contribute to their own regular growth [63, 64]. We assumed that this diet might have an impact on the variations in the phytochemical contents of sericin across the three species.

### Silk sericin antioxidant capacity

#### DPPH free-radical scavenging ability

Various studies have reported that sericin possess free-radical scavenging activity against DPPH radical [51, 63, 65, 66]. The substance’s capacity to scavenge DPPH radicals can be interpreted as its antioxidant activity [67]. As a result, it is not unexpected to conclude that all the silkworm species had electron donors that interacted with free radicals transforming them into more stable compounds. Several studies have reported that phytochemicals found on silkworm layers play a role in the radical scavenging ability of silk sericin [2, 7, 68]. Previous studies have examined the relationship between total phenolic content and antioxidant properties of various plants and the findings indicated that phenolic compounds play a significant role in their antioxidant activities [69, 70]. Therefore, the differences in antioxidant activities of sericin from the three species could be attributed to their differences in polyphenolic content. The catechol group in phenolic compounds structures neutralizes free radicals while the antioxidant ability of flavonoids is attributed to its capacity in donating an electron that neutralizes free radicals [71].

#### Silk Sericin antibacterial activity

Sericin from all the three species had considerable antibacterial activities with *S*. *ricini* showing the best activity against *K*. *pneumonia*, *P*. *aeruginosa*, *S*. *aureus* and *E*. *coli*. The findings of our study are consistent with other research that has indicated sericin exhibits antibacterial action against both Gram-positive and Gram-negative bacteria. For instance, in a study conducted by Senakoon and co-authors [72] to determine the antibacterial activity of Eri powder recovered via different degumming methods against *E*. *coli* and *S*. *aureus*, 90-minute Na_2_CO_3_ degummed Eri sericin effective dose was at 30 μg. This was more effective against Gram-negative *E*. *coli* than the 60-minute water degummed Eri sericin which had an effective dose of 40 μg. Remarkably, only the 60-minute water degummed Eri sericin was seen to inhibit *S*. *aureus*. To determine the antibacterial activity of the wild silkworms (*G*. *postica*, *G*. *rufobrunnea*, and *Argema mimosa*) in South Africa, Manesa *et al* [65] reported that all the three species had considerable antibacterial activity against Gram-positive bacteria (*B*. *subtilis*, *S*. *aureus and S*. *epidermidis*). Moreover, they came to the conclusion that inhibitory zones would not rise above a dosage of 10 mg sericin/ml.

It has been suggested that polyphenols, through their antibacterial and antibiotic-modulating properties, have chemo-preventive and therapeutic benefits. Furthermore, studies have indicated that flavonoids exhibit strong antibacterial activity against both Gram-negative and Gram- positive microorganisms [73, 74]. Thus, we believe that *S*. *ricini’s* highest phytochemical content and antioxidant activity could be the reason for its greater efficacy against *P*. *aeruginosa*, *K*. *pneumonia*, *S*. *aureus*, and *E*. *coli*. In addition to phytochemicals, silk sericin contains cationic amino acid side chains which are influenced by the presence of NH_3_^+^ groups, thus positively charged [65]. Conversely, the surface of bacterial cell walls is negatively charged, a phenomenon brought about by the cell wall outer envelope containing ionized phosphoryl and carboxylate substances [75, 76]. These interactions cause disruptions in the stiffness and stability of the cell, which leads to the leakage of the bacteria’s proteinaceous components and other cellular contents. [65] thus inhibiting their growth and development [77]. This opposite charge phenomenon has been demonstrated in several studies [28, 78, 79]. Thus, we propose that interactions between sericin’s polycationic side groups and the negative charges on the surface of bacterial cell walls may be responsible for the compound’s antibacterial action [65]

Besides polycationic interactions, sericin antibacterial activity could be influenced by its molecular weight [80]. According to reports, the recovery technique utilized in this work, that is (HTHP), causes sericin hydrolysis and thus reduce its molecular weight [81]. Sericin with lower molecular weights could penetrate directly through the bacterial walls and form anionic complexes within the bacterial cells [82]. This results in interruptions of the normal bacterial cellular physiology and eventually causes bacterial death [82, 83]. According to this study, sericin possesses strong antibacterial activities and can be applied in biomedical field to formulate wound hydrogels for wound management.

## Conclusion

Sericin from all the three species are potential sources of natural antioxidants which can be attributed to the presence of phytochemicals. Additionally, they have considerable broad-spectrum antibacterial activity and thus it can be applied as an antibacterial agent. Considering interspecies variation, *S*. *ricini* had higher activities compared to *B*. *mori* and *G*. *postica*. Thus, it’s evident from this study that sericin from the three species has potential medical uses and thus we recommend further research to be undertaken on the application of silk sericin in biomedical applications especially on wound management.

## Supporting information

S1 File (PDF)

S2 File (PDF)

S3 File (PDF)
