# Supplementary material for: Evaluating antibacterial and antioxidant properties of sericin recovered from cocoons of Bombyx mori, Gonometa postica and Samia ricini in Kenya
Source: PLoS One. 2024 Dec 31;19(12):e0316259. doi: 10.1371/journal.pone.0316259 (PMC11687748; doi:10.1371/journal.pone.0316259)
Supplement: S1 File — (PDF) [file pone.0316259.s001.pdf]

Species	Length	Chloramp|	Streptomy	Oxytetracyc	Lengh/Chlo	lengh/Strep	Lengh/ Oxy	Oxytetracyc
S. aureus								
Gonometa	9.874	25.671	15.328		38.46364	64.41806		
Gonometa	10.21	25.671	15.328		39.77251	66.61013		
Gonometa	9.686	25.671	15.328		37.73129	63.19154		
Samia rici	8.859	25.671	15.328		34.50976	57.79619		
Samia rici	11.741	25.671	15.328		45.73643	76.59838		
Samia rici	11.955	25.671	15.328		46.57006	77.99452		
Bombyx m	8.783	25.671	15.328		34.2137	57.30037		
Bombyx m	9.186	25.671	15.328		35.78357	59.92954		
Bombyx m	8.887	25.671	15.328		34.61883	57.97886		
S. pyogene								
Gonometa	12.67	27.761		25.136	45.63957		50.40579	
Gonometa	11.848	27.761		25.136	42.67858		47.13558	
Gonometa	8.783	27.761		25.136	31.63791		34.94192	
Samia rici	8.262	27.761		25.136	29.76118		32.86919	
Samia rici	9.101	27.761		25.136	32.7834		36.20703	
Samia rici	9.628	27.761		25.136	34.68175		38.30363	
Bombyx m	13.531	27.761		25.136	48.74104		53.83116	
Bombyx m	12.359	27.761		25.136	44.51929		49.16852	
Bombyx m	13.578	27.761		25.136	48.91034		54.01814	
K. pneumuniae								
Gonometa	10.442	13.698	14.614	19.61	76.23011	71.45203	53.24834	
Gonometa	10.589	13.698	14.614	19.61	77.30326	72.45792	53.99796	
Gonometa	10.078	13.698	14.614	19.61	73.57278	68.96127	51.39215	
Samia rici	13.454	13.698	14.614	19.61	98.21872	92.06241	68.60785	
Samia rici	12.505	13.698	14.614	19.61	91.2907	85.56863	63.76849	
Samia rici	12.344	13.698	14.614	19.61	90.11535	84.46695	62.94748	
Bombyx m	11.988	13.698	14.614	19.61	87.51643	82.03093	61.13208	
Bombyx m	11.424	13.698	14.614	19.61	83.39904	78.17162	58.25599	
Bombyx m	11.893	13.698	14.614	19.61	86.82289	81.38087	60.64763	
P.aeuriginosa								
Gonometa	11.525		19.591	13.909		58.82803	82.86002	
Gonometa	11.028		19.591	13.909		56.29115	79.28679	
Gonometa	11.811		19.591	13.909		60.28789	84.91624	
Samia rici	12.832		19.591	13.909		65.49946	92.25681	
Samia rici	12.98		19.591	13.909		66.25491	93.32087	
Samia rici	13.351		19.591	13.909		68.14864	95.98821	
Bombyx m	8.258		19.591	13.909		42.15201	59.37163	
Bombyx m	8.821		19.591	13.909		45.02578	63.41937	
Bombyx m	9.382		19.591	13.909		47.88934	67.45273	

E.coli					
Gonometa	10.059	16.325	20.951	61.61715	48.01203
Gonometa	9.146	16.325	20.951	56.0245	43.65424
Gonometa	9.521	16.325	20.951	58.32159	45.44413
Samia rici	8.873	16.325	20.951	54.35222	42.3512
Samia rici	9.402	16.325	20.951	57.59265	44.87614
Samia rici	9.132	16.325	20.951	55.93874	43.58742
Bombyx m	7.903	16.325	20.951	48.41041	37.72135
Bombyx m	8.363	16.325	20.951	51.22818	39.91695
Bombyx m	8.038	16.325	20.951	49.23737	38.36571

:line
