# Supplementary material for: Evaluating antibacterial and antioxidant properties of sericin recovered from cocoons of Bombyx mori, Gonometa postica and Samia ricini in Kenya
Source: PLoS One. 2024 Dec 31;19(12):e0316259. doi: 10.1371/journal.pone.0316259 (PMC11687748; doi:10.1371/journal.pone.0316259)
Supplement: S2 File — (PDF) [file pone.0316259.s002.pdf]

Species	TPC	TFC
B.mori	343.4247	220
B.mori	314.6575	253.3333
B.mori	333.8356	336.6667
G. postica	273.5616	220
G. postica	340.6849	220
G. postica	248.9041	253.3333
S. ricini	731.0959	586.6667
S. ricini	857.1233	653.3333
S. ricini	751.6438	570

Species	Antioxidant
B.mori	19.45525292
B.mori	24.90272374
B.mori	25.68093385
B.mori	26.84824903
B.mori	26.07003891
G. postica	33.46303502
G. postica	36.57587549
G. postica	36.18677043
G. postica	35.40856031
G. postica	38.52140078
S. ricini	34.24124514
S. ricini	43.19066148
S. ricini	42.0233463
S. ricini	43.19066148
S. ricini	39.68871595

Species	%yield
B.mori	21.22
B.mori	13.76
B.mori	28.48
B.mori	36.16
B.mori	46.36
B.mori	39.88
B.mori	29.22
B.mori	31.62
B.mori	39.56
B.mori	12.48
S.ricini	6.06
S.ricini	6.3
S.ricini	0.86
S.ricini	7.6
S.ricini	5.02
S.ricini	9.08
S.ricini	5.78
S.ricini	2.56
S.ricini	8.94
S.ricini	2.76
G. postica	9.96
G. postica	12.84
G. postica	9.12
G. postica	9.44
G. postica	12.48
G. postica	11.7
G. postica	12.1
G. postica	10.88
G. postica	7.72
G. postica	13.92
