# Supplementary material for: Evaluating antibacterial and antioxidant properties of sericin recovered from cocoons of Bombyx mori, Gonometa postica and Samia ricini in Kenya
Source: PLoS One. 2024 Dec 31;19(12):e0316259. doi: 10.1371/journal.pone.0316259 (PMC11687748; doi:10.1371/journal.pone.0316259)
Supplement: S3 File — (PDF) [file pone.0316259.s003.pdf]

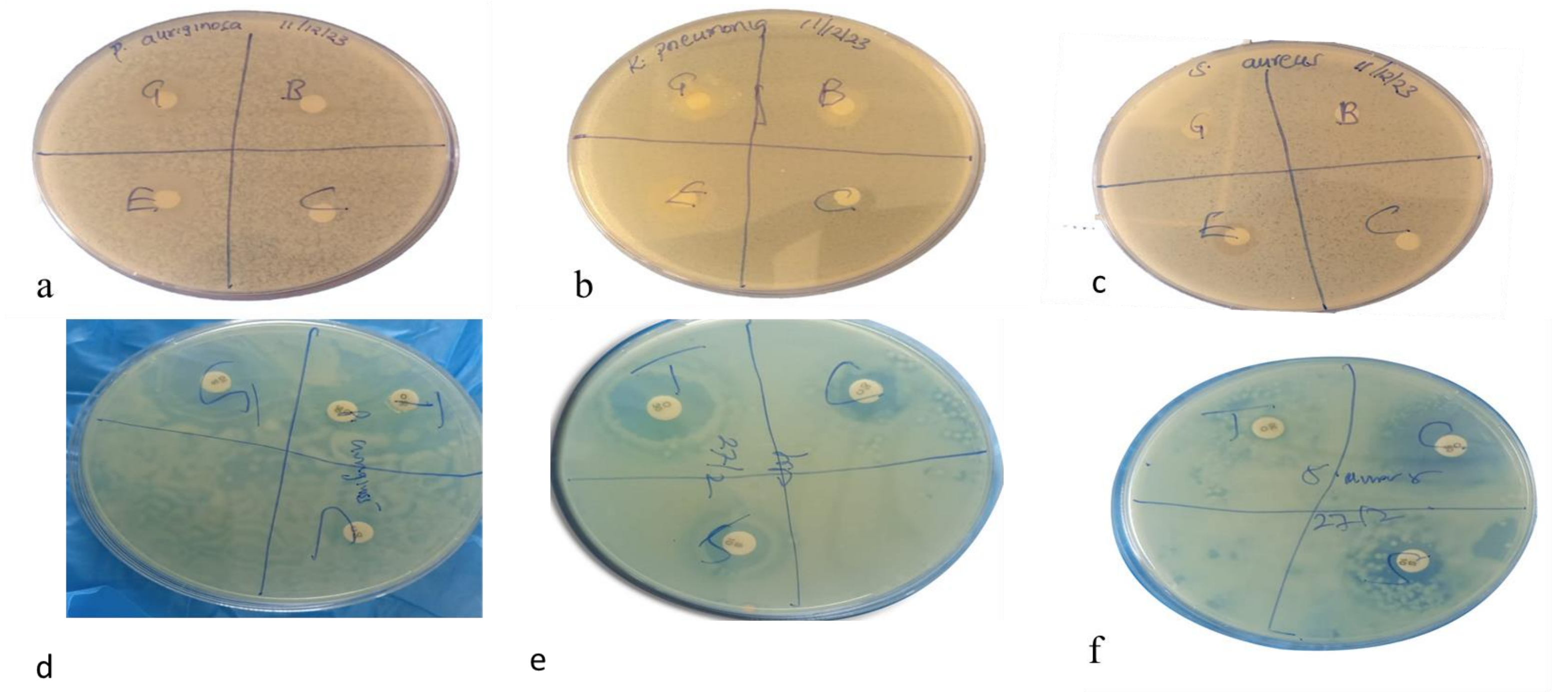

Representatives of bacterial zones of inhibition displayed by distinct silk sericin and antibacterial discs against various bacteria.

G- *G. postica*, B- *B. mori* , E- *S. ricini* C- Negative control on petri dishes a, b and c

T- oxytetracycline disc, S- Streptomycin and C- chloramphenicol on dish d, e and f.

a and d – *P. aeruginosa*

b and e – *K. pneumonia*

c and f- *S. aureus*
